# Microbiome in Neuroblastoma: A Virgin Island in the World of Onco-Microbiome

**DOI:** 10.3390/cells14151218

**Published:** 2025-08-07

**Authors:** Ashwath Keshav Giri, Poorvi Subramanian, Loganayaki Periyasamy, Sivaroopan Aravindan, Natarajan Aravindan

**Affiliations:** Department of Physiological Science, College of Veterinary Medicine, Oklahoma State University, Stillwater, OK 74078, USA; ashwath.giri@okstate.edu (A.K.G.); poorvi.subramanian@okstate.edu (P.S.); loganayaki.periyasamy@okstate.edu (L.P.); roopanaravindan@gmail.com (S.A.)

**Keywords:** microbiome, neuroblastoma, gut microbiota, dysbiosis, pediatric cancer

## Abstract

The composition of the gut and/or tumor microbiome has been intricately involved in the onset of carcinogenesis, tumor progression, therapy response, and patient outcomes in diverse solid cancers. The microbiome type, composition, and their metabolome have been functionally implicated in the multifarious cellular processes, transformation, proliferation, tumor immune evasion, cellular migration, etc. Despite such compelling evidence on the role of microbiome interactions in cancer, the realization of their role in neuroblastoma (NB), the deadly extracranial tumor in infants is few and fragmentary. This review comprehends the composition, diversity, and significance of microbiota in human health. Further, this review discusses the microbiota composition, their mode of action, and their signaling flow through and cellular processes in diverse cancers including NB. Precisely, this study for the first time has realized the functional relevance and clinical significance of the gut and tumor microbiome for NB. Interestingly, large cohort clinical and preclinical in vivo models of NB realized the following: gut microbiota predicts the risk for NB; postnatal (and or not maternal transmission) microbiome rearrangements; gut microbial effect on NB pathogenesis; tumor-altering gut microbial composition; microbial composition predicts treatment outcomes in NB; prebiotic remedies for stabilizing NB-associated microbial rearrangements; microbial composition in tumor-infiltrating microbiota predicts NB outcomes.

## 1. Microbiome

The human body functions as a complex ecosystem, inhabited by trillions of microorganisms collectively known as the microbiota or microbes. In the natural environment, microorganisms establish communities, with each species having a distinct ecological function. Microorganisms, despite their small size and invisible nature, significantly contribute to human health and overall well-being [1]. The microbiome is an entire collection of microorganisms—including bacteria, viruses, fungi, and archaea, along with their genetic material—which interact with their surrounding environment [2]. The definition of “microbiota” refers to microorganisms, while “microbiome” covers the entire habitat, including the microbes, their genomes, and the related environmental factor [3]. Research on the human microbiome has revealed the presence of symbiotic bacteria living on various body surfaces, including the skin, airways, urinary system, teeth, and epithelial lining of the gastrointestinal tract [4].

### 1.1. Composition of Microbiota

**Figure 1:**
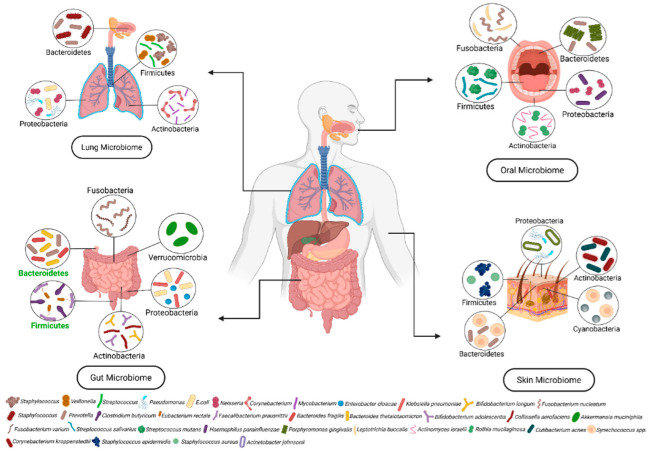
**Visual Representation of the Human Microbiome Across Body Sites.** Human-associated microbial communities exhibit remarkable spatial diversity, with distinct bacterial populations colonizing specific anatomical sites. The lung microbiome, though less dense, primarily contains *Firmicutes*, *Bacteroidetes*, *Proteobacteria*, and *Actinobacteria*. The oral microbiome, the second largest microbial community, mainly includes *Firmicutes*, *Proteobacteria*, *Bacteroidetes*, *Actinobacteria*, and *Fusobacteria*. The human gut microbiome is primarily composed of six dominant phyla—*Firmicutes*, *Bacteroidetes*, *Proteobacteria*, *Actinobacteria*, *Verrucomicrobia*, and *Fusobacteria*—with *Firmicutes* and *Bacteroidetes* being the most abundant. The skin microbiome harbors site-specific microbes from *Cyanobacteria*, *Proteobacteria*, *Actinobacteria*, *Bacteroidetes*, and *Firmicutes*.

The population of microbiota varies between anatomical regions, such as the lungs, oral, gut, and skin [1] (Figure 1). Its composition is influenced by extrinsic factors, such as age, nutrition, and environmental circumstances, which are essential for maintaining homeostasis [2]. The human gut microbiome, characterized by distinct individual microbial compositions, typically consists of six principal phyla: Actinobacteria (1–10%), Bacteroidetes (20–40%), Firmicutes (40–60%), Fusobacteria (<1%), Proteobacteria (1–5%), and Verrucomicrobia (<1%). Among these, Bacteroidetes and Firmicutes constitute 90% of the gut microbiome [3]. The oral microbiota is regarded as the second biggest microbial community in humans, with the major bacterial phyla being Actinobacteria (5%), Bacteroidetes (18%), Firmicutes (38%), Fusobacteria (9%), and Proteobacteria (30%), collectively constituting about 96% of the oral microbiome, and the remaining 4% is composed of several minor phyla including spirochaetes, chlamydiae, tenericutes, and euryarchaeota [4]. The human skin microbiota comprises a unique assemblage of microorganisms, including Actinobacteria (40–50%), Bacteroidetes (5–10%), Cyanobacteria (3%), Firmicutes (25–30%), and Proteobacteria (15–20%) [5]. The core lung microbiota is primarily composed of microorganisms from the phyla Actinobacteria (5–10%), Bacteroidetes (30–40%), Firmicutes (25–35%), and Proteobacteria (20–30%), while minor phyla such as Fusobacteria, Chloroflexi, Verrucomicrobia, and TM7 (Saccharibacteria) typically comprise less than 5% [6]. Its composition is mainly influenced by three fundamental factors: (1) the invasion of microorganisms, (2) the eradication of microbes, and (3) the replication rates of microorganisms [7]. Invasion of microbes occurs through various mechanisms, including microaspiration, via which oropharyngeal and upper respiratory bacteria, such as Streptococcus pneumoniae, invade the lungs [8]. Airborne inhalation of microorganisms, such as *Haemophilus influenzae* and *Mycobacterium tuberculosis*, along with hematogenous dissemination in the bloodstream, can contribute to microbial colonization in the lungs [9]. Direct exposure through medical procedures, including ventilators, can introduce stable microbial populations like *Klebsiella pneumoniae* [10]. Microbes can easily be eliminated by several types of defenses such as mucociliary clearance, where inhaled microbes get trapped in mucus and are expelled by cilia [11]. The replication of microorganisms in the lungs is mediated by nutrient availability, host immune responses, and environmental conditions, with Firmicutes proliferating in high-fat diets and Bacteroidetes linked to fiber-rich diets [12]. Besides bacteria, the microbiome includes fungi, viruses, archaea, and protozoans, which contribute to intricate ecological interactions with the microbial ecosystem. Fungi, although less studied than bacteria, contributes to the gut microbiome, with prevalent species such as Candida, Saccharomyces, Malassezia, and Cladosporium [13]. Along with bacteria and fungi, the human gut microbiota also comprises viruses (*Norovirus* and *Rotavirus*), phages (T4 phage and Lambda phage), and archaea (*Methanobrevibacter smithii*) [14].

### 1.2. Traits of Microbial Diversity

Microbial diversity is established at birth and is continuously modulated throughout life, with diet exerting the greatest influence, followed by the mode of delivery and other environmental factors. Diet affects the gut microbiota, with sustained dietary habits determining its composition and diversity. Fiber-rich, plant-based diets and fermented items enhance the proliferation of beneficial bacteria and the synthesis of short-chain fatty acids (SCFAs) [15]. The mode of childbirth significantly influences the newborn’s microbiome. During vaginal birth, infants are exposed to their mother’s vaginal and intestinal microbiota, promoting a diverse and advantageous initial microbiome with necessary bacteria such as *Bifidobacterium* and *Lactobacillus* facilitating the development of the immune system and metabolic functions [16]. In contrast, babies delivered via caesarean section are frequently protected with maternal skin-associated bacteria, predominantly *Staphylococcus* species, resembling skin microbiota more than gut microbiota [17]. This variation in microbial exposure emphasizes the role of the birth mode in forming the infant’s microbiome and consequently increases the risk of metabolic and immune-related illnesses later in life [18]. In the initial phase of life (0–12 months), the microbiome undergoes rapid development, with breastfeeding being crucial as it supplies advantageous bacteria such as Bifidobacterium and human milk oligosaccharides (HMOs), which develop the proliferation of beneficial bacteria, mainly *Bifidobacterium* and *Lactobacillus*, enhancing the immune system and digestive health [19]. In infants (1–3 years), a varied diet fosters the development of bacteria essential for immunity and digestion, resulting in a microbiome like that of adults, which is characterized by increased levels of Firmicutes and Bacteroidetes. By the end of this period, the gut microbiome attains greater stability, establishing a foundation for future health [20]. During childhood (4–12 years), the microbiome is more diverse and has an adult-like community structure [21]. A balanced adult microbiome includes Firmicutes, Bacteroidetes, Actinobacteria, and Proteobacteria, essential for metabolism, immune function, and disease resistance [22]. Adolescence is an important stage that features hormonal changes, and dietary and lifestyle modifications, and increased exposure to environmental bacteria influences the microbiota and indicates the continuous maturation of the immune system [23]. (Table 1) illustrates the evolution of the microbial community across the human lifespan, from birth to adulthood [24]. Environmental factors influence the human microbiome via both macro- and microenvironments. Specific exposures, such as smoking and alcohol consumption, can alter the oral and gut microbiomes, thereby increasing health risks. Toxicants in the built environment, such as volatile organic compounds (VOCs) like benzene and formaldehyde found in paints, cleaning products, and air fresheners, can disrupt microbial balance and influence health, including the development of asthma [25].

In addition to chronological age and developmental stages, numerous extrinsic and intrinsic determinants substantially influence the composition of the gut microbiota. Among these, the use of antibiotics and medications can profoundly alter the gut microbiome, reducing microbial diversity and eradicating beneficial taxa [26]. Genetics also play a crucial role in determining the initial microbial colonization and can influence long-term microbial composition, though environmental factors often exert a stronger effect [27]. Geographic location, sanitation practices, and socio-economic factors are also critical variables, as individuals from rural or traditional communities frequently display enhanced microbial diversity compared to their urban counterparts [28]. Regular physical activity is consistently associated with increased gut microbial diversity and metabolic benefits [29], while disruptions to sleep patterns and circadian rhythms may negatively impact microbiome. Environmental exposures such as tobacco use and alcohol intake modify the gut microbiota with potential health ramifications, while exposure to domestic toxicants, including volatile organic compounds from consumer products, can disturb microbial equilibrium and has been associated with conditions such as asthma [30]. Although age and dietary habits are principal determinants, a diverse array of environmental, lifestyle, and physiological factors continually modulate the trajectory and health implications of the human gut microbiome.

### 1.3. Microbiota in Human Health

A well-balanced microbiome enhances human health by aiding in food digestion and nutrient absorption, regulating immune system functions, influencing mental health via the gut–brain axis, contributing to metabolic activities, and defending the body against harmful pathogens [31]. The microbiome helps in regulating the immune system by interacting with immune cells and maintaining homeostasis. Helpful microbial metabolites such as SCFAs support immunological tolerance and protect against infections [32], while SCFAs generated by gut bacteria also influence cognitive function via the gut–brain axis; however, dysbiosis (an imbalance in the gut microbiota) disrupts the gut–brain connection, ultimately resulting in mental health issues such as anxiety and depression [33]. In addition to its protective and regulatory roles, the microbiome also impacts disease susceptibility, including cancer. Microbial pathogens such as *Fusobacterium nucleatum* and *Escherichia coli* (*E. coli*) facilitate carcinogenesis, whereas beneficial metabolites like butyrate exhibit anti-carcinogenic properties [34,35]. The microbiome further influences cancer susceptibility via modulating inflammation, DNA damage, and metabolic processes, enhancing responses to immunotherapy and chemotherapy [36]. Children with malignancies clearly have different microbiota compositions than healthy children. Children with leukemia, for example, exhibit altered bacterial patterns and reduced microbial diversity [37].

Dysbiosis in the microbiome has been associated with a broad spectrum of disorders. It plays a significant role in the pathogenesis of chronic inflammatory conditions, mainly inflammatory bowel disease (IBD), which is due to an imbalance in the composition of microorganisms, which results in a decrease in the number of beneficial bacteria and an increase in the number of pathogenic microorganisms, thereby promoting chronic inflammation [38]. In IBD, a decrease in the abundance of anti-inflammatory bacteria (such as *Faecalibacterium prausnitzii*) and an increase in pro-inflammatory taxa aggravate mucosal immune activation and tissue injury [39]. A similar association has been shown between dysbiosis and metabolic syndrome and obesity, all of which are disorders that are defined by altered energy extraction and elevated inflammatory responses, both of which contribute to the course of disease [40]. Gut microbiota in obesity exhibits increased Firmicutes-to-Bacteroidetes ratios and an enhanced capacity for energy harvest from the diet, promoting fat deposition and low-grade inflammation [41]. In type 2 diabetes, there is a reduced abundance of butyrate-producing bacteria and an increased proportion of Gram-negative bacteria, which collectively promote inflammation and impair glucose metabolism [42]. In addition, changes in the microbiome can cause disruptions in immunological tolerance, which can lead to the development of allergic disorders, autoimmune diseases, and insulin resistance in diabetics [43]. Dysbiosis can influence the gut–brain axis, exacerbating neuroinflammation in neurodegenerative disorders such as Alzheimer’s disease, Parkinson’s disease, Multiple Sclerosis, Huntington’s Disease, Autism Spectrum Disorder, and Chronic Fatigue Syndrome [44] (Table 2). Host genetic characteristics, antibiotic use, immunological response, nutrition, drugs, infections, chronic diseases, and environmental exposures are all factors that might lead to microbial dysbiosis [45,46]. The human microbiome has become a critical factor in understanding the progression of diseases and their responses to therapy, shedding light on the broader implications for health [47].

**Table 2:** Delineates disease-specific gut microbiota compositional shifts, underlying host and environmental factors contributing to microbial dysbiosis, and the resultant pathophysiological effects on neuroinflammatory and neurodegenerative processes via the gut–brain axis.

Disease	Microbiome Involved	Factors Contributing to Dysbiosis	Effect of Dysbiosis	Reference
Parkinson’s disease	↑ *Akkermansia*, *Lactobacillus* ↓ *Prevotella*, *Roseburia*	Genetic traits, pesticide exposure, antibiotic use, diet	Exacerbates neuroinflammation via gut–brain axis	[48]
Alzheimer’s disease	↑ *Escherichia/Shigella*, *Bacteroides* ↓ *Faecalibacterium prausnitzii*	Aging, poor nutrition, inflammation, chronic disease	Promotes neuroinflammation and amyloid-beta deposition	[49]
Multiple Sclerosis	↑ *Methanobrevibacter smithii*, *Akkermansia* ↓ *Clostridia*,	Vitamin D deficiency, antibiotics, environmental factors	Alters immune regulation and gut permeability	[50]
Huntington’s Disease	↑ *Proteobacteria*, ↓ *Lactobacillus*	Genetic mutation, oxidative stress, altered nutrition	Disrupts metabolic and neuroimmune homeostasis	[51]
Autism Spectrum Disorder	↑ *Clostridia*, *Desulfovibrio* ↓ *Bifidobacterium*	Cesarean birth, early antibiotic exposure, formula feeding	Impairs neurodevelopment via microbial metabolite imbalance	[52]
Chronic Fatigue Syndrome	↑ *Enterobacteriaceae* ↓ *Faecalibacterium*, SCFA-producing bacteria	Viral infections, chronic stress, gut permeability	Drives systemic inflammation and immune dysfunction	[53]

## 2. Microbiome in Cancer

**Figure 2:**
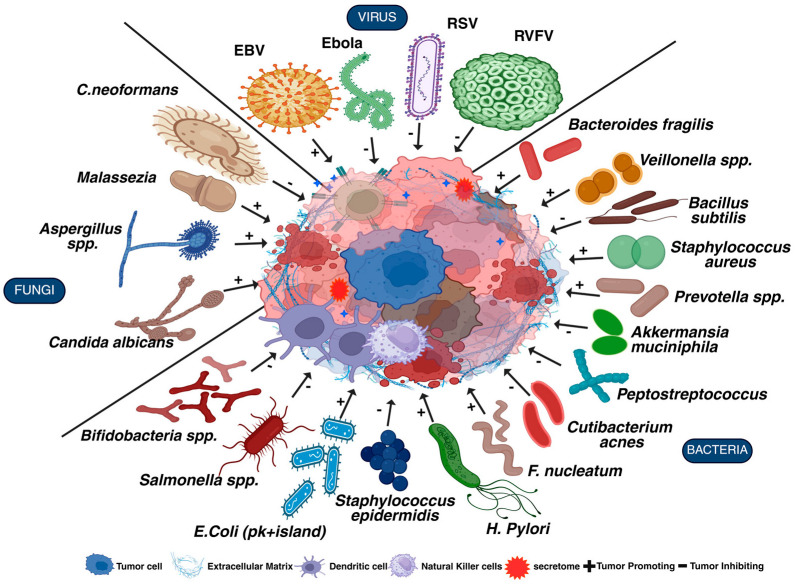
**Interaction of Microbial Pathogens with Cancer Cells.** This schematic illustration depicts various microbial pathogens, including viruses, bacteria, and fungi, that may interact with cancer cells. The viral pathogens include Epstein–Barr Virus, Respiratory Syncytial Virus, Ebola virus, and Rift Valley Fever Virus, which may influence the tumor microenvironment through immune modulation and oncogenic properties. The bacterial pathogens consist of *F. nucleatum*, *Veillonella*, *Staphylococcus epidermidis*, *Bacillus subtilis*, *Micrococcus*, *H. pylori*, *Escherichia coli*, *Salmonella*, and *Bifidobacteria*, *Peptostreptococcus*, *Cutibacterium acnes*, *Akkermansia muciniphila*, *Staphylococcus aureus*, *Prevotella*, which may contribute to tumor progression and inflammation, or possess probiotic and therapeutic roles. The fungal pathogens, including Aspergillus, *C.neoformans*, *Candida albicans*, and *Malassezia*, can alter immune responses and affect tumor dynamics. These microbial interactions with cancer cells may have significant implications in tumor development, immune evasion, and microbiome-based therapeutic strategies.

Recent research highlights the complex interaction between the microbiome and cancer, showing that microbial populations are essential in cancer development and progression [54], influencing carcinogenesis through mechanisms such as immune system modulation, microbial metabolite synthesis, and influence on cancer treatments [55]. About 20% of human cancers are linked to microbes [56] and understanding these connections could pave the way for new prevention strategies and treatments that target microbial influences on cancer. The microbiome aids in cancer development through inflammation and genotoxic agents. A modification in the microbiome elevates pro-inflammatory cytokine levels, thereby disrupting the immune system and increasing the risk of various cancers, such as pancreatic and colorectal cancer, which not only promote tumor growth but also hinder the effectiveness of treatments [57]. Currently, eleven organisms—seven viruses (*Hepatitis B*, type 1 *human T cell lymphotropic virus*, *Epstein–Barr virus*, *Hepatitis C*, *Kaposi sarcoma herpesvirus*, *human immunodeficiency virus-1*, *human papilloma viruses*), three platyhelminths (*Schistosoma haematobium*, *Clonorchis sinensis*, *Opisthorchis viverrini*), and one bacterium (*Helicobacter pylori*)—have been explicitly identified as unique causes of cancer in humans [58] (Figure 2). These microorganisms play a significant role in cancer progression including neuroblastoma (NB) through several mechanisms, such as activation of B cell differentiation, disruption of normal cell cycle, inflammation, angiogenesis, cytokine production, cell proliferation and transformation, metabolic shift, invasion, metastasis, stromal reprogramming, desmoplasia, genomic instability, tissue remodeling, DNA damage, immune hyperactivation [59] (Table 3). T-cell dysregulation (in EBV and HTLV infection), and direct oncogenesis induced by Hepatitis virus and KSV in hepatocellular carcinomas and Kaposi sarcoma [60]. The gut microbiome influences the development, progression, and therapeutic outcomes of both gastrointestinal (e.g., colorectal, gastric, esophageal, pancreatic) [61] and non-gastrointestinal cancers (e.g., lung, breast, prostate, ovarian, skin) [62].

**Table 3:** Overview of microbe-associated signaling pathways implicated in various cancers, detailing specific microbial species, the molecular mechanisms they influence, and their resultant effects on tumorigenesis, immune modulation, and cancer progression.

Cancer	Microbes	Pathway	Mechanism	Effects	Reference
Colorectal Cancer	*Fusobacterium nucleatum*	Wnt/β-catenin	FadA from *F. nucleatum* activates β-catenin via E-cadherin binding	Promote proliferation and tumor initiation	[63]
	*Enterotoxigenic Bacteroides fragilis*	NFκB/STAT3	BFT toxin and LPS stimulate chronic inflammation, IL-6 upregulates STAT3	Drives inflammation and immune evasion	[64]
	*E. coli* (*pks+*) *island*	PI3K/AKT/mTOR	Colibactin and ROS activate PI3K/Akt Promotes tumorigenesis Alters tumor suppressor genes TP53 and proto-oncogenes KRAS	Enhance survival, angiogenesis	[65]
	*F. nucleatum*, *E. coli*	TLR/MyD88/MAPK	Microbial ligands activate TLRs → MyD88 → MAPK cascade	Inflammatory signaling, cytokine production	[66]
Gastric Cancer	*H. pylori* (*CagA*)	SHP-2/Ras/ERK	CagA protein activates NFκB (direct injection into epithelial cells); LPS induces inflammation loss of polarity and hyperproliferation	Promotes cell proliferation and transformation	[67]
	*H. pylori*, *Peptostreptococcus*	NFκB/STAT3	IL-6, TNFα driven by *H. pylori* and others → survival signaling	Chronic inflammation and immune modulation	[68]
	*H. pylori* (*VacA*)	PI3K/AKT	SCFAs and VacA promote survival and immune suppression	Cell survival and metabolic shift	[69]
	*F. nucleatum* *Peptostreptococcus*,	EMT-related (Snail, Twist)	Inflammation triggers EMT programs via NFκB and others	Invasion and metastasis	[70]
Esophageal Cancer	*P. gingivalis*, *F. nucleatum*	NFκB/IL-6/STAT3	Chronic exposure to *P. gingivalis* LPS and *F. nucleatum* leads to NFκB activation → IL-6 secretion → STAT3 phosphorylation via TLR4 signaling Drives cell survival, angiogenesis, and immune evasion	Inflammation-driven survival, growth, and angiogenesis	[71]
	*Candida albicans*	EGFR/STAT3	*Candida albicans* and oral dysbiosis increase EGF, HER2 receptor kinase, TGF-α (EGFR ligand expression) → EGFR activation → STAT3-driven survival and proliferation	Proliferation, inhibition of apoptosis	[72]
	*Candida albicans*, *Prevotella*, *Veillonella*	TLR/MyD88/MAPK	TLR2/4 recognize PAMPs from fungi and Gram-negative bacteria → activate MyD88 → downstream MAPKs (ERK, JNK, p38) → cytokine storm and inflammation	Immune modulation and inflammation	[73]
Pancreatic Cancer	*Malassezia*	KRAS/MAPK	Mutant KRAS is central to PDAC. Microbiota-driven inflammation (e.g., IL-1β, TNFα) enhances KRAS downstream signaling (ERK, MEK) Increased proliferation and survival	Drives oncogenesis	[74,75]
	*E. coli*, *Malassezia*	Hedgehog (Shh/Gli)	Microbial imbalance can dysregulate Shh/Gli signaling in the tumor microenvironment, leading to excessive stroma formation, reducing drug delivery and enhancing immune exclusion	Stromal reprogramming and desmoplasia	[76]
	*F. nucleatum*, *Malassezia*, *E. coli*	TLR/MyD88	Bacterial and fungal components (LPS, β-glucans) bind TLRs → MyD88-dependent signaling → NFκB/MAPK activation → inflammation Macrophage reprogramming to pro-tumor M2 phenotype	Inflammation and immune evasion	[77]
Breast Cancer	*Firmicutes*, *Proteobacteria*	Estrogen Metabolism	Altered microbiome composition increases β-glucuronidase activity → deconjugates estrogen glucuronide → promotes reabsorption of active estrogen	Increased risk of ER+ breast cancer	[78]
	*Bacteroides* spp.	Estrobolome	Produces β-glucuronidase enzyme that deconjugates estrogens in the gut → estrogen re-enters circulation (enterohepatic recycling)	Elevated estrogen levels promote ER+ breast cancer	[78,79]
	*E. coli*, *S. aureus*	NFκB, STAT3	Induce ROS and inflammatory cytokines Promote DNA double-strand breaks Activate survival and proliferation pathways	Promotes tumorigenesis via genomic instability and inflammation	[80]
Lung Cancer	*Akkermansia muciniphila*	TLR2–IL-10 axis	Stimulates TLR2 → promotes anti-inflammatory cytokine IL-10 Enhances gut barrier integrity and Treg induction	Protectiv—reduces inflammation and promotes immune surveillance	[81]
	*Prevotella* spp, *Veillonella*,	TLR/MyD88/NFκB	Bacterial PAMPs (e.g., LPS) bind TLRs (TLR2, TLR4), activates MyD88 → NFκB pathway, cytokine release (IL-6, TNFα) → chronic inflammation	Promotes inflammation, DNA damage, immune suppression → tumor initiation and progression	[82]
	*Bifidobacterium* spp.	TLR9/IFN-γ signaling	Stimulates TLR9 on dendritic cells → Increases IFN-γ, CD8+ T cell activation Enhances antigen presentation	Enhances anti-tumor immunity, reduces immunosuppressive microenvironment	[83]
	*Haemophilus*, *Streptococcus*,	MAPK/ERK	TLR signaling and microbial cytokines activate MAPK cascade Activates ERK, p38 → gene expression for proliferation	Increases proliferation, survival, and tissue remodeling favorable for tumor development	[84]
Skin Cancer	*Staphylococcus aureus*	TLR2/TLR4	Lipoteichoic acid and peptidoglycan activate TLR2/TLR4 → MyD88-dependent NFκB activation → IL-6, IL-1β secretion Produces toxins that induce reactive oxygen species (ROS) → oxidative stress	Enhances tumor progression and immune evasion and DNA damage, genomic instability	[85]
	*Staphylococcus epidermidis*	6-HAP-mediated Inhibition	Produces 6-HAP (6-N-hydroxyaminopurine) → inhibits DNA polymerase activity → reduces DNA synthesis in tumor cells	Suppresses tumor growth and exhibits protective effects	[86]
	*Cutibacterium acnes*	TLR2/NFκB	Activates TLR2 on keratinocytes → NFκB activation → pro-inflammatory cytokine release Induces chronic inflammation and oxidative stress	Promotes DNA damage and tumor initiation	[87]
Brain Cancer	*Bacteroides fragilis*	Kynurenine/AHR Pathway	Alters tryptophan metabolism → increases kynurenine → activates aryl hydrocarbon receptor (AHR) in brain tissues	Favors glioblastoma progression	[88,89]
	*Clostridium* spp	Epigenetic Modulation via SCFAs	Produces butyrate → inhibits histone deacetylases (HDACs) → promotes apoptosis and DNA repair in glial cells	Opposes tumor proliferation in gliomas	[90,91]
	Prevotella spp.	Th17/IL-17	Promotes IL-17-producing Th17 cells via mucosal stimulation → enhances systemic inflammation and disrupts blood–brain barrier (BBB) integrity	Facilitates immune cell infiltration and may promote glioma invasiveness	[92]
Neuroblastoma	*Bacteroides fragilis*	STAT3/NFκB	Accumulations of myeloid-derived suppressor cells and inhibition of dendritic cell differentiation	Supports tumor progression	[93]

### 2.1. Gut Microbiome in Cancer Development

While both GI and non-GI cancers are influenced by microbial dysbiosis, the nature of this influence differs significantly. In GI cancers, the microbiome exerts a direct effect on tumorigenesis through physical contact with the mucosal epithelium, promoting inflammation, genotoxicity, and epithelial barrier disruption. In contrast, non-GI cancers are affected more indirectly, with the microbiome modulating systemic immune responses and hormone metabolism and influencing the efficacy of immunotherapies. Additionally, non-GI cancers are influenced by microbial metabolites and immune signaling originating from distant sites like the gut or oral cavity. These mechanistic differences underscore the critical need for precision microbiome-targeted strategies tailored to the anatomical site and tumor microenvironment for effective cancer prevention, diagnosis, and treatment. The direct and indirect (or otherwise the GI and non-GI cancer) effects on the composition, function, and determinants of the microbiome are discussed below.

#### 2.1.1. Gastrointestinal Cancer (GI)

Colorectal cancer (CRC) is one of the most extensively studied malignancies affected by the gut microbiome. The colon harbors a high density of microbial populations, and dysbiosis is strongly associated with CRC initiation, progression, and prognosis. CRC malignancies have been found to harbor pathogenic bacteria, including *Fusobacterium nucleatum*, which promotes inflammation, alters the immunological microenvironment, and enhances tumor aggressiveness by activating Toll-like receptors (TLRs) [94]. *F. nucleatum* exerts its oncogenic effects adhesin FadA, which attaches to E-cadherin on epithelial cells, triggering the β-catenin/Wnt signaling pathway [63]. This cell proliferation pathway is often dysregulated in colorectal cancer. *F. nucleatum* stimulates Wnt signaling by stabilizing β-catenin, which translocates to the nucleus and expresses genes that promote cell growth and survival, promoting tumorigenesis [95]. Simultaneously *F. nucleatum* develops a pro-tumorigenic microenvironment by recruiting immune cells and promoting the synthesis of pro-inflammatory cytokines like IL-6 and TNFα, hence supporting tumor growth and enabling immune evasion [96]. CRC is marked by distinct microbial alterations, including an overabundance of *E. coli*, and *Bacteroides fragilis*, along with a reduction in beneficial bacteria like *Roseburia* and other butyrate-producing bacteria [97]. *E. coli*, *Enterococcus faecalis*, and *H. pylori* are among pathogenic bacteria that cause persistent inflammation that promote carcinogenesis [98]. *E. coli* strains with the polyketide synthase (pks) island generate colibactin, a genotoxin causing DNA damage that alters tumor suppressor genes TP53 and proto-oncogenes KRAS [65]. Genomic instability caused by this mutagenic effect favors early adenoma formation and cancer development. Enterotoxigenic Bacteroides fragilis generates *Bacteroides fragilis* toxin (BFT), which degrades E-cadherin, disturbing the tight junctions [64]. This improves cell permeability and triggers the Wnt/β-catenin and Nuclear factor kappa light chain enhancer of activated B cell (NFκB) signaling pathways, promoting inflammation, epithelial hyperplasia, and tumor development [99]. Beneficial gut bacteria such as *Faecalibacterium prausnitzii* and *Roseburia* generate SCFAs such as butyrate, which have anti-cancer activity [100]. By inhibiting histone deacetylases (HDAC), butyrate promotes expression of tumor suppressor genes and preserves intestinal epithelial integrity [101]. Conversely, bacteria like *Bifidobacterium* and *Lactobacillus* reduce inflammation and provide cancer-preventive effects [102]. *H. pylori* significantly contributes to the development of gastric cancer (GC) by affecting the gastric epithelium through the release of toxins like CagA (Cytotoxin-associated gene A) and VacA (Vacuolating cytotoxin A) [103]. CagA disrupts epithelial junctions and modifies cell polarity by interfering with important signaling pathways like SHP-2 and Ras/ERK, thereby affecting cell behavior and promoting uncontrolled growth [67]. Beyond *H. pylori*, other bacteria, including *Lactobacillus*, *Streptococcus*, *Peptostreptococcus*, *Fusobacterium*, *Prevotella*, and *Veillonella*, become more prominent when H. pylori colonization decreases due to antibiotics, immunological responses, and microbial competition [104]. Patients with esophageal cancer exhibit an increased presence of firmicutes, Actinobacteria, Streptococcus, and Actinomyces in the microbiome, along with a decrease in Bacteroidetes and Prevotella [105]. Higher levels of Firmicutes and Actinobacteria, along with Streptococcus and Actinomyces, cause persistent inflammation and oxidative stress, leading to DNA damage [106]. In contrast, a decrease in Bacteroidetes and Prevotella weakens the defensive mechanism, exacerbating inflammation, leading to the progression towards esophageal adenocarcinoma [107]. Pancreatic cancer has been progressively linked to bacterial translocation from the gastrointestinal tract to pancreatic tissues [108], where specific microorganisms, *Fusobacterium nucleatum* and *Porphyromonas gingivalis*, identified in pancreatic tumors and ductal tissues are colonized [109]. These bacteria contribute to immune evasion by inhibiting T cell responses and activating inflammatory pathways through TLRs, which drive oncogenic signaling and tumor growth [110]. Additionally, *Malassezia*, a fungal component, translocates to the pancreas, triggering the complement cascade and further driving tumor growth [74].

#### 2.1.2. Non-Gastrointestinal (Non-GI) Cancer

Comprising malignancies in organs like the breast, lungs, skin, and brain, non-gastrointestinal cancers constitute a major worldwide health burden and greatly influence cancer-related morbidity and mortality [111]. Changes in the local microbiome—in particular a rise in Firmicutes and Proteobacteria—have been linked to breast carcinogenesis and estrogen metabolism [112]. Evidently the gut microbiome influences breast cancer risk mainly by regulating estrogen metabolism [113]. One major pathway is estrogen metabolism via the ‘estrobolome,’ where the bacterial species (e.g., *Bacteroides* spp.) generate β-glucuronidase, an enzyme deconjugating estrogen glucuronide discharged into the gut [79]. Particularly in postmenopausal women, this reactivation allows estrogens to be reabsorbed into the bloodstream through enterohepatic circulation, thereby increasing estrogen levels and the risk of estrogen receptor-positive (ER+) [78]. *Clostridium* spp. helps to offset this mechanism by generating butyrate, which lowers inflammation, improves regulatory T cells, and acts as an HDAC inhibitor to support tumor cell death and DNA repair [114]. By activating TLRs to balance cytokines and increase immunoglobulin A (IgA), *Lactobacillus* spp. helps immunity by encouraging dendritic and NK cells, playing a protective role against breast cancer development and progression [115]. Alterations in microorganisms—especially a decrease in beneficial bacteria like *Akkermansia muciniphila* and *Bifidobacterium* spp., and an increase in inflammation-related genera like *Prevotella* spp.—have been linked to lung cancer development and compromised immunotherapy response [81]. A decrease in *Akkermansia muciniphila was* found to strengthen the gut barrier and stimulate Th1 immune responses by promoting dendritic cell maturation and interferon-γ production, thereby improving response to anti-PD-1 immunotherapy [116]. *Bifidobacterium* spp. activates dendritic cells and secretes pro-immune cytokines to increase T cell priming and immune checkpoint inhibitor activity, helping tumor recognition and clearance [117]. Conversely, an increase in *Prevotella* spp. is associated with low-grade chronic inflammation and immune dysregulation [118]. It promotes Th17-type immune responses and the synthesis of IL-6 and IL-17, which helps in creating a tumor-supportive microenvironment [119]. Microbial dysbiosis in skin cancer—including melanoma and non-melanoma forms—is found to be a source of persistent inflammation, fostering carcinogenesis. In melanoma, the gut microbiota is essential in determining the reaction to immunological checkpoint blockade (ICB) treatments [120]. Particularly pathogens like *Staphylococcus aureus* have been accompanied by a higher risk of skin malignancies, underscoring the important function of the skin microbiome in cancer development [121]. Melanoma patients with poor responses to immunotherapy are enriched *with Prevotella* spp. and *Ruminococcus* spp., which foster immune suppression by growing regulatory T cells and raising anti-inflammatory cytokines [122]. The microbiome influences brain cancers, such as gliomas (tumors originating from glial cells), by modifying immune responses in the central nervous system (CNS) via gut-derived bacteria, which affects tumor proliferation and therapy resistance [88].

### 2.2. Direct and Indirect Effects of Microbiome

To understand the unique roles of microbes in tumor growth and progression, it is crucial to distinguish between direct and indirect microbial effects on cancer. Direct effects occur when microbial species or their products interact with cancer cells or their immediate microenvironment, altering processes such as cell signaling, DNA integrity, or immune evasion. For instance, gut bacteria can create metabolites that promote apoptosis or decrease tumor cell development [123]. In contrast, indirect effects arise when the microbiome influences host systems, particularly the immune, metabolic, or neuroendocrine systems, which in turn affect cancer development, diagnosis, or treatment outcomes [124]. The GI microbiome interacts directly through colonization, digestion, and immune regulation [125] (Table 4), while the non-gastrointestinal microbiome (skin, oral, etc.) interacts indirectly via immunological, metabolic, and neuroendocrine mechanisms [126].

**Table 4:** Microbial metabolites, associated microbes, and their roles in cancer progression and immune modulation.

Cancer	Microbial Metabolites	Microbes Involved	Role of Microbial Metabolites in Cancer	Reference
Colorectal cancer	SCFA-butyrate	*Clostridium butyricum*	Butyrate enhances immune responses and inhibits tumor progression by altering T cell differentiation and stemness.	[127]
	Ferroptosis Inhibitors-Lactate	*E. coli*, *Klebsiella*	Inhibit ferroptosis to prevent iron-dependent oxidative damage and promote CRC cell survival and growth.	[128,129]
	Indole-3-propionic acid	*Lactobacillus*, *Bifidobacterium*	Indole-3-acetate modulates immunological responses via the gut homeostasis-maintaining aryl hydrocarbon receptor (AhR) and enhances mucosal barrier integrity.	[130]
	Tryptophan Metabolites- kynurenine	*E. faecalis*	Inhibit immune responses and contribute to tumor immune evasion.	[131]
	Phenylacetic Acid (PAA)	*Bacteroides*, *Lactobacillus*	Affects tumor microenvironment and metabolic pathways by modulating immune responses for cell proliferation.	[123]
Gastric cancer	N-nitroso Compounds (NOCs)	*E. coli*, *Enterococcus faecalis*	Nitrosamines are mutagenic, generating DNA adducts that induce mutations and genetic instability.	[132]
	tryptophan, arginine	*Lactobacillus*, *streptococcus*	Upregulated in neoplastic tissues; facilitate tumor proliferation and immune evasion through metabolic reprogramming.	[133]
Esophageal cancer	Perfluorooctanoate	*Clostridium leptum*	Increased PFOA levels influenced by *C. leptum* are linked to heightened EC risk. Endocrine disruptor	[134]
	SCFAs	*Phascolarctobacterium*, *Fusobacterium nucleatum*	SCFAs can modulate inflammation and support tumor growth by promoting lipid synthesis and maintaining epithelial proliferation	[107]
	Lipopolysaccharides	*Fusobacterium nucleatum*	Promotes chronic inflammation, leading to epithelial damage and carcinogenesis and activates TLR4/NFκB signaling pathway, increasing IL-6, TNFα	[135]
Pancreatic cancer	Trimethylamine N-oxide	*Clostridium sporogenes*, *Anaerococcus hydrogenalis*	Enhances anti-tumor immunity; administration in PDAC-bearing mice reduced tumor growth and activated effector T cell responses	[136]
	Indole-3-acetate	*Enterococcus faecalis*, *Lactobacillus* spp.	Suppress the anti-tumor activity by inducing immunosuppressive tumor-associated macrophages	[137]
Breast cancer	SCFA- butyrate, propionate, acetate	*Eubacterium rectale*, *Clostridium perfringen*, *Faecalibacterium prausnitzii*	SCFA act as HDAC inhibitors, inducing apoptosis, cell cycle arrest, and epigenetic changes and modulate IL-10 and TGF-β.	[138]
	Trimethylamine N-oxide	*Clostridiales*, *Faecalibacterium*, *Ruminococcaceae*	Induces ferroptosis or pyroptosis in tumor cells and promotes anti-tumor immunity	[138]
Lung cancer	kynurenine, indoles	*Clostridium sporogenes*, *Lactobacillus*	Regulate pulmonary immune microenvironment via aryl hydrocarbon receptor signaling	[139]
	Secondary bile acids	*Clostridium*, *Eubacterium*	Influence lung immunity via gut–lung circulation and improve immunotherapy outcomes	[139]
Skin cancer	Lipoteichoic acid	*Staphylococcus epidermidis*	Inhibits UV-induced skin tumor formation via TLR2 signaling.	[140]
	Phenol-soluble modulins	*Staphylococcus aureus*, *Cutibacterium acnes*	Promote inflammation and immune evasion, contributing to squamous cell carcinoma development	[140]
Brain cancer	Polyunsaturated fatty acids	*Alistipes*, *Bacteroides*	Dysregulated PUFA metabolism leads to neuroinflammation, which is a shared mechanism in glioma and brain tumors	[141]
	Arachidonic acid, Phenylacetic acid	*Bacteroides*, *Clostridium scindens*	Promotes neuroinflammation and amyloid-beta aggregation	[142]

#### 2.2.1. Microbial Metabolites (Direct Effect)

One significant class of microbial metabolites involved in cancer progression is the secondary bile acids [143]. *Clostridium scindens* synthesize deoxycholic acid (DCA) through the 7α-dehydroxylation of primary bile acids in hepatocellular carcinoma (HCC) [144]. DCA generates oxidative stress in the liver, leading to ROS accumulation that damages DNA strand breaks, disrupts chromosomal integrity, and promotes mutations in tumor suppressors genes like *TP53* [145]. Additionally, DCA activates inflammatory pathways, such as NFκB and STAT3, that promote cell proliferation, angiogenesis, and immune evasion [146]. Microbial genotoxins underline how direct genomic changes and inflammatory signaling pathways in the microbiome contribute to carcinogenesis. In addition to the genotoxic effects, gut bacteria produce microbial metabolites including SCFAs, folate, and B vitamins. These metabolites function as epigenetic controllers, influencing gene expression and contributing to cancer development [147]. SCFAs, particularly butyrate, modify DNA methylation and histone modifications, leading to changes in the gene expression without changing the DNA structure [148]. This process can either drive cancer progression or prevent its occurrence, depending on whether it activates or suppresses oncogenes and tumor suppressor genes [149].

#### 2.2.2. Reactive Oxygen and Nitrogen Species (Direct Effect)

Highly reactive molecules known as reactive oxygen species (ROS) and reactive nitrogen species (RNS) are very important in the development and progression of cancer [150]. They are produced as natural byproducts of cellular metabolism, particularly through mitochondrial activity and immune responses. However, they have the potential to contribute to cellular damage, inflammation, and the development of cancer when their production becomes excessive or unregulated [151]. *Enterococcus faecalis* can generate ROS, including H_2_O_2_ and O_2_^−^ which damage DNA [152], proteins, lipids, and biological structures. This damage may result in DNA mutations or strand breaks, undermining the integrity of genetic material and consequently heightening the risk of cancer, especially colorectal cancer [153]. ROS and RNS not only induce DNA damage but also activate oncogenic pathways that promote inflammation, cellular proliferation, and cancer progression. The NFκB signaling pathway is one of the most significant pathways triggered by ROS. Under normal circumstances, NFκB is strongly regulated, but excessive ROS causes continuous NFκB activation, releasing pro-inflammatory cytokines including Tumor Necrosis Factor-alpha (TNFα), Interleukin-6 (IL-6), and Interleukin-1 beta (IL-1β) [154]. To that end, we have shown that amplification of ROS could be a sensing mechanism for the activation of the signaling cascade that influences self-fate, including transformation, death, or survival. Previously our studies have shown that an increase in ROS can trigger NFκB signaling that in turn leads to feedback through transactivation of SOD2 and controls the switch that directs self-fate [155]. Eventually we unearthed how the ROS-activated NFκB could initiate and maintain TNFα, IL-1α, cMYC, and SOD2-dependent autocrine and/or paracrine cellular response. In general, NFκB is believed to be an immediate early response, transcriptional regulator, whose activation upon a cellular insult function is like an umbrella response. However, our sequential studies convey the critical requirements of NFκB sustained (2nd/3rd phase activation) through unique systemic cyclic feedbacks for steering specific functions [156]. For instance, ROS-triggered NFκB-mediated TNFα-dependent cyclic feedback steers cellular survival; NFκB-triggered MMP9-mediated ERK-dependent [156] cyclic feedback sustains NFκB for endorsing cellular migration and NFκB-mTOR signaling feedback for clonal expansions [155]. These cytokines not only promote cancer cell survival and multiplication but also cause chronic inflammation. Activation of NFκB also promotes the expression of genes linked to angiogenesis and metastases, therefore accelerating the growth of tumors [157]. Furthermore, ROS and RNS stimulate the signal transducer and activator of the transcription 3 (STAT3) pathway, an important regulatory mechanism for inflammation, by increasing immune evasion by overexpressing genes that allow malignancies to escape immune surveillance. Chronic activation of STAT3 increases cancer cell survival, provides resistance to cell death, and promotes metastasis [158].

#### 2.2.3. Immune Modulation (Indirect Effect)

The immune system is essential for tumor surveillance, maintaining an essential balance between anti-tumor and pro-tumor responses. Immune function is greatly dependent on the microbiome, which regulates both innate and adaptive immune systems and which helps to eliminate tumors or contribute to cancer progression [159]. The immune system is essential for tumor surveillance, with the innate immune system comprising macrophages, neutrophils, and dendritic cells acting as the primary defense. Among these, macrophages can exhibit dual roles: the M1 type combats tumors, while the M2 type supports tumor growth and progression [160]. However, dysbiosis in the microbiome can disturb this equilibrium, leading to activation of TLRs which drive macrophages toward the M2 phenotype, thereby enhancing tumor growth through the stimulation of angiogenesis and modification of adjacent tissues to aid cancer progression [161]. The adaptive immune system, including T cells, B cells, and NK cells, is critical for the elimination of malignant cells. Dysbiosis leads to a disruption in the equilibrium between effector T cells, which are responsible for promoting tumor rejection. Consistently regulatory T cells (Tregs) lead to the expansion of Tregs in the TME, suppressing the anti-tumor immune response [162].

#### 2.2.4. Cancer Therapy Modulation

Beyond transformation, carcinogenesis, and tumor progression, microbiomes are important for modulating cancer therapy. Particularly, in non-GI cancers, microbial populations found in places like the oral cavity and skin have been proven to influence the effectiveness of immunotherapy, chemotherapy, and radiation treatment among other cancer treatments [163]. Immunotherapy has emerged as a transformative approach in the treatment of cancer, particularly through checkpoint inhibitors such as Programmed Cell Death Protein 1 (PD-1) and Cytotoxic T-Lymphocyte Antigen 4 (CTLA-4), which have altered cancer care by utilizing the immune system of the body to target and destroy tumor cells in diverse cancers such as melanoma, lung cancer, bladder cancer, and other types of cancer [164]. Patients with melanoma who have higher specific skin and gut microbiota species respond better to anti-PD-1 therapy, suggesting that microbiome composition enhances the effectiveness of immunotherapy and immune response [165]. *Faecalibacterium prausnitzii* and *Bacteroides thetaiotaomicron* affect the efficacy of immune checkpoint inhibitors like anti-PD-1 by enhancing the anti-tumor immune response by influencing the effector T cell, thereby modulating systemic immune activation [166]. Dysbiosis in the oral, cutaneous, or respiratory microbiome can impair immune responses and diminish the efficacy of chemotherapy [167]. Studies indicate that oral microorganisms alter cytokine production and immunological responses, thereby influencing therapeutic outcomes [168]. Oral dysbiosis in breast cancer patients is correlated with unsatisfactory chemotherapy response, probably due to heightened inflammation and modified drug metabolism [169], which eventually influences the immune system’s response to treatment by modulating inflammation, altering metabolic pathways, and thus impacting the effectiveness of cancer therapies, diminishing their efficacy.

## 3. The Role of Gut Microbiota in Pediatric Cancer: Implications for Immune Modulation, Dysbiosis, and Therapeutic Interventions

Childhood malignancies are the primary cause of disease-related morbidity and mortality in children in the United States and around the globe, after accidents [170]. Among childhood cancers, acute lymphoblastic leukemia (ALL) accounts for around 25% of all pediatric cancers [171], with leukemias overall being the most prevalent, followed by brain and CNS tumors. Within the pediatric solid tumors, NB and Wilms tumor are the most frequently diagnosed cancers [172]. The gut microbiota plays a pivotal role in the formation and functioning of the immune system [173]. Growth and activity of the immune system depend on the gut microbiota, particularly in childhood cancer, as inadequate immune response can promote cancer growth and progression [174]. The early-life microbiome significantly influences immunological responses through its interaction with gut-associated lymphoid tissue (GALT) [175], which promotes immune tolerance, modulates inflammation, and aids in the development of critical immune cells, including T cells [176], regulatory T cells (Tregs), and antigen-presenting cells, which is essential for differentiating between self- and non-self-antigens [177]. High levels of inflammatory cytokines, such as IL-6, TNFα, and IL-1β, are often observed in pediatric malignancies. These cytokines can alter the composition of the gut microbiota, leading to dysbiosis [178]. Dysbiosis can contribute to gut barrier dysfunction and increased permeability, which facilitates the entry of harmful microbial products, such as lipopolysaccharides (LPS), into the systemic circulation. This mechanism is particularly relevant in pediatric cancers, where the gut barrier and immune system are still developing. Unlike adults, children have a more immature epithelial lining and a microbiome in the process of being established, making them more vulnerable to systemic inflammation caused by microbial translocation. Disruptions during this developmental window, due to chemotherapy, antibiotics, poor nutrition, or hospitalization, can have more profound and lasting effects, promoting tumor progression more significantly than in adult malignancies. Additionally, gut bacteria, which play a crucial role in immune system development from infancy, can influence immune responses. These responses can either promote or hinder oncogenesis [179]. This higher gut permeability triggers systematic inflammation, worsens immune system malfunction, and accelerates cancer progression.

Pediatric brain tumors—especially medulloblastomas and gliomas—represent a considerable share of childhood malignancies, with medulloblastomas the most common malignant brain tumor in children [180] and glioblastoma contributing to notable morbidity and death. Neuroinflammation, tumor growth, and immune responses in pediatric brain tumors are influenced by the gut–brain axis [181], an emerging field of study that indicates a critical interaction between gut microbiota and CNS pathology. In addition to immune signaling and microbial metabolites, recent evidence suggests that the gut microbiota may influence levels of brain-derived neurotrophic factor (BDNF), a key modulator of neurodevelopment, synaptic plasticity, and cognitive function [182]. Alterations in the gut microbiome, especially during early life, can reduce BDNF expression in brain regions such as the hippocampus and cortex, potentially impairing hippocampal neurogenesis and microglial regulation [183]. In pediatric brain tumors like medulloblastoma and glioma, reduced BDNF levels have been associated with tumor-associated neuroinflammation, which may exacerbate disease progression [184]. Thus, gut microbiota-induced modulation of BDNF represents another critical axis through which microbial dysbiosis can influence CNS pathology in pediatric cancers. The gut microbiome can influence the immune system’s capacity to identify and react to tumor cells via microbial metabolites, such as LPS and tryptophan metabolites, which travel the blood–brain barrier (BBB) and impact neuroimmune signaling [185]. SCFAs, such as butyrate and propionate, have shown the ability to modulate neuroinflammation by enhancing the production of anti-inflammatory cytokines, particularly Interleukin-10 (IL-10), and maintaining the integrity of the gut epithelium and BBB [186]. The gut microbiota is crucial for the maturation and activation of microglia, the resident immune cells of the CNS, with microbial signals necessary for development [187]. In addition to this, the gut microbiota profoundly affects the CNS via multiple processes, including microglial activity, neurotransmitter synthesis, and possibly increasing neuroinflammation linked to brain cancers [188]. Therefore, dysbiosis can lead to chronic microglial activation, fostering a pro-tumor environment in gliomas [189]. Similarly, lymphomas, comprising Hodgkin’s lymphoma (HL) and non-Hodgkin’s lymphoma (NHL), are prevalent pediatric malignancies that affect the immune system [190]. The gut microbiome is essential for the modulation of the systemic immune system, as it influences the development of lymphoid tissue, the differentiation of immune cells, and chronic inflammation—key factors in lymphomagenesis [191]. The absence of beneficial bacteria, including Firmicutes and Bacteroidetes, can further exacerbate systemic inflammation and disrupt immune homeostasis, thereby contributing to the development of lymphoma [192]. Although the effect of gut bacteria on solid cancers such NB and Wilms tumor is yet unknown, early-life microbial exposure can be suggested as a preventive measure against NB development [193]. NB, a tumor of the sympathetic nervous system, arises from neural crest cells (NCCs) [194] and its progression is influenced by immune system interactions [195] modulated by gut microbiota. Initial immunological conditioning by the gut microbiota helps the immune system distinguish between self- and non-self-antigens, while breastfeeding supports this process by promoting beneficial bacteria like Bifidobacterium and Lactobacillus [196], which enhance immune development and may reduce NB risk through increased IgA production.

Early infancy is known as the “critical period” due to the increased susceptibility of the microbiome to alteration, where the gut flora eventually becomes stable throughout the course [197]. In pediatric patients with AML, Bacteroides and Bifidobacterium levels were considerably decreased six weeks post-leukemia treatment, when compared to control subjects, despite the recovery of other genera [198]. This reveals that children receiving cancer treatment during this critical period develop microbial dysbiosis even after the last course of therapy. Dysbiosis is primarily caused by the depletion of microbiomes that are beneficial to health or by an increase in bacteria that are harmful to health [45]. Host genetics shape gut microbiota composition, diversity, and stability. In childhood cancer patients, immune responses and microbiota composition are determined by genetic predisposition. Some genetic variants linked to immunological function, such as TLRs, can change microbial interactions and cause dysbiosis by means of mutations in pattern recognition receptors [199]. Genetic causes of reduced microbial diversity could contribute to increased gut permeability, sometimes referred to as “leaky gut,” which allows bacterial components to enter systemic circulation and causes chronic inflammation [200]—a known risk factor for children malignancies including leukemia. Antibiotic administration significantly contributes to gut microbial dysbiosis in the general population by reducing microbial diversity and altering the composition of beneficial bacteria [201]. One of the most important consequences of antibiotic treatment is the reduction of helpful, commensal bacteria such as *Bifidobacterium* and *Lactobacillus*, which helps in the regulation of immune responses, including the promotion of anti-inflammatory cytokines, in which the immune system distinguishes between beneficial and harmful pathogens [202]. The immune system’s capacity to regulate inflammation and sustain tolerance declines when antibiotics decreases the bacterial populations [203]. Pediatric cancer patients receiving rigorous therapies frequently suffer from malnutrition because of appetite, sickness, changed metabolism, and gastrointestinal side effects, which can significantly affect gut microbiota and overall immune system function [204]. More specifically, a diet low in prebiotic-rich foods, such as oligosaccharides, can impair the growth of beneficial gut bacteria. In such conditions, *Bifidobacterium*, *Lactobacillus*, and other fiber-fermenting bacteria that are essential for producing SCFAs may be destroyed [205]. More precisely, vitamins can drastically change the balance of gut flora and weaken the immune systems in pediatric cancer patients [206]. Vitamin A is important for keeping epithelial integrity, immune cell growth, and mucosal immunity, and its deficiency can impair the gut barrier, allowing pathogenic microbes to invade and causing dysbiosis [207]. Similarly, low vitamin D intake or absorption brought on by adverse effects from medication may aggravate dysbiosis and raise the risk of cancer development [208]. Because of their immunocompromised state and intensive therapies, pediatric cancer patients sometimes suffer hospital-associated infections (HAIs) [209]. While sanitized, hospitals limit exposure to different natural bacteria, therefore lowering gut microbiota diversity and allowing dangerous bacteria to flourish—which cause dysbiosis [210]. This imbalance worsens the immune system, increasing the risk of infections. Exposure to environmental contaminants such as heavy metals, pesticides, and toxins affects the gut microbiota in children receiving cancer therapy, fostering dangerous bacteria, compromising the gut barrier, and promoting immunological dysfunction and inflammation, which delays recovery [211].

Given the significant consequences of dysbiosis, various techniques are being explored to re-establish gut microbial equilibrium in children receiving cancer therapy. Especially in pediatric cancer patients, prebiotics and probiotics have garnered curiosity for their ability to restore gut flora composition and function [212]. Prebiotics are non-digestible dietary fibers that nourish and promote the growth of beneficial gut bacteria, hence promoting a stable microbiome and enhancing overall immune health; probiotics are advantageous living microorganisms that aid in restoring gut microbiota balance and augmenting immune function [213]. *Lactobacillus rhamnosus* and *Bifidobacterium breve* probiotic supplements have been demonstrated to restore microbial diversity in leukemia patients enduring intensive chemotherapy [214], thereby rebalancing gut flora and improving overall health outcomes. Additionally, probiotics also lower the gastrointestinal side effects related to cancer treatment. Two prevalent and debilitating side effects of chemotherapy, diarrhea and mucositis, greatly affect the quality of life in childhood cancer [215]. Studies have demonstrated that probiotic supplements may significantly decrease the severity and length of chemotherapy-induced diarrhea, therefore enhancing treatment tolerance and general well-being [216]. Conversely, prebiotic supplementation—especially with galactooligosaccharides (GOS) and fructooligosaccharides (FOS)—has been shown to enhance Bifidobacterium and Lactobacillus, while lowering the intestinal inflammation in pediatric cancer patients [217]. SCFAs which control immune responses, lower systemic inflammation, improve nutrient absorption, and strengthen gut barrier integrity are produced when prebiotics are fermented by gut bacteria [218]. This helps immune recovery following chemotherapy and helps to prevent malnutrition—a problem in pediatric cancer patients suffering appetite loss and gastrointestinal toxicity. Prebiotics and probiotics are a good way to manage gut flora imbalances, reduce therapy-related issues, and boost the immune system in pediatric cancer patients [219]. In addition to prebiotics and probiotics, other emerging strategies restoring the gut microbial balance include synbiotics (a combination of prebiotics and probiotics), postbiotics (non-viable microbial products or metabolites that confer health benefits), and fecal microbiota transplantation (FMT), which has shown promise in re-establishing microbial diversity in immunocompromised patients [220]. Furthermore, dietary interventions rich in fiber, polyphenols, and fermented foods are being investigated for their ability to support a healthy microbiome and enhance treatment outcomes [221]. These approaches, alongside prebiotic and probiotic supplementation, represent a growing toolkit for managing dysbiosis and improving immune resilience in pediatric oncology. Despite the microbiome restoration techniques in pediatric cancer, recent studies indicate an association between gut microbiota and the progression, immune response, and treatment results in particular malignancies, including NB, which is associated with immunological dysregulation and inflammation, affected by the gut microbiome [222]. Further in-depth investigations on such relationships could result in the development of novel medicines that improve immune function and treatment efficacy.

## 4. Neuroblastoma

NB, an embryonal neuroendocrine tumor arising from neural crest progenitor cells [223], accounts for 9.1% of pediatric cancer deaths. NB originates from sympathoadrenal progenitors generated from the neural crest during embryonic development and possesses distinct clinical and biological features. NCCs, a unique class of pluripotent cells, arise early in the embryonic development, a trait of the origin of NB [224]. Originating from the ectodermal layer, these cells migrate to parts of the body, developing into a wide spectrum of tissues, including neurons, glial cells, and components of the peripheral nervous system [225]. Despite intensive multi-modal clinical therapy (IMCT) [226,227], more than half of high-risk phenotypic patients will relapse with hematogenous metastasis [228]. The treatment of high-risk illness is rare due to the disease’s heterogeneity, resistance, and poor hematological reserve, resulting in less than 10% five-year overall survival and 2% 10-year survival, compared with 38–71% for low-risk disease [229,230]. Typically, high-risk diseases are characterized by numerous genetic and molecular abnormalities [231]. Somatic MYCN amplification occurs in around 20% of NB cases and is independently associated with advanced stage and poor outcomes. MYCN amplification occurs in just 25–35% of high-risk NB, while 65–75% are classified as MYCN non-amplified (MYCN-na) [232,233]. The IMCT for high-risk NB comprises an (i) induction phase alternating high-dose chemotherapy, (ii) consolidation phase intensifying chemotherapy, radiation, and stem cell transplantation, and (iii) maintenance phase utilizing retinoids, immunotherapy, and cytokines. The first relapse takes >18 months and decreases significantly subsequently due to genetic and molecular rearrangements in undifferentiated tumorigenic NCCs that mediate NB progression [227]. Our recent investigations using a mouse model of PD showed that aggressive CSC-like NB cells exhibit reversible and adaptive plasticity, determining the evolution of NB [234]. Given the varying survival outcomes associated with different risk groups, staging plays a crucial role in determining disease severity and guiding appropriate treatment strategies. NB is categorized according to two important staging systems: the International Neuroblastoma Risk Group Staging System (INRGSS) and International Neuroblastoma Staging System (INSS). The INRGSS (Table 5) determines the stage of NB based on the imaging result, while the INSS (Table 6) uses surgical results to determine the cancer’s stage [235]. In NB, epigenetics plays a critical role [236]. Alterations in genes such as TP53 and MYCN [237] contribute to the progression and severity of the disease by playing a key role in cancer growth and propagation.

The microbiome composition varies significantly throughout different stages of NB, potentially affecting disease progression. In patients with early-stage or low-risk NB, the microbiome is associated with immune homeostasis and tumor progression [238]. Bacteroidetes and Firmicutes are two major phyla in the healthy gut that are essential for maintaining homeostasis and regulating inflammatory responses, thereby enhancing anti-tumor activity [239]. A balanced Firmicutes-to-Bacteroidetes ratio contributes to the optimal gut barrier function, preventing the spread of pro-inflammatory bacterial endotoxins [240]. In addition, the presence of Lactobacillus and Bifidobacterium, two well-known probiotic genera, promotes gastrointestinal health and immune regulation by modulating cytokines and causing anti-inflammatory effects [241]. These probiotics increase regulatory T cell (Treg) function, facilitating immunological tolerance and decreasing excessive immune activation linked to tumor growth [242].

Considering the NCCs origin of NB and its intricate connection with the immune system, the microbiome, which significantly modulates both innate and adaptive immunity, affects tumor growth and therapeutic results. Certain beneficial gut bacteria enhance the anti-tumor immune responses by stimulating essential immunological components. *Bifidobacterium* species enhance the dendritic cell (DC) functionality via the stimulator of interferon genes (STING) signaling pathway [243] thereby promoting antigen presentation and the activation of cytotoxic T cells [244] essential against NB, where vigorous T cell activation is imperative to combat the immunosuppressive TME (Table 7). In addition to T cells, NK cells are also crucial for the immune response against NB by detecting and eliminating cancer cells without prior sensitization. NB cells often evade NK cell-induced apoptosis by downregulating major histocompatibility complex class I (MHC-I) molecules and secreting immunosuppressive cytokines (Transforming growth factor-beta (TGF-β), IL-6, IL-10, and prostaglandin E2 (PGE2)) [245]. The gut microbiome, particularly Lactobacillus species, enhances NK cell cytotoxicity by modulating cytokine synthesis. This results in increased levels of interferon-gamma (IFN-γ), TNFα, and interleukin-2 [246] and upregulating activating NK cell receptors such as natural killer group 2 member D (NKG2D), which is crucial for targeting NB cells, especially in conjugation with monoclonal antibody therapy like dinutuximab [247]. Another beneficial bacterium, *Faecalibacterium prausnitzii*, produces butyrate, which plays an important role in immune homeostasis and the mitigation of chronic inflammation [248]. This production of butyrate assists in maintaining a balanced immune response and supports immune-mediated tumor suppression in NB. Although beneficial microbes enhance anti-tumor immunity, certain pathogenic bacteria promote immunosuppressive TME, thereby facilitating immune evasion and tumor progression. NB tumors evade immune surveillance by recruiting regulatory T cells (Tregs) and myeloid-derived suppressor cells (MDSCs), which collectively suppress anti-tumor immune responses and foster immunosuppressive TME [249].

Despite the increasing interest in the microbiome’s function in many malignancies, the relationship between NB and the microbiome remains relatively unexplored. A comprehensive examination of the thus far documented studies sheds some, yet significant, light on the association between NB and the microbiome. These limits significantly recognize the gap in the field, especially for these deadly developmental tumors. Owing to the clear demarcation of clinically favorable immune hot NB with commendable survival compared to the progressive, immune cold NB with negligible survival, the understanding of the microbiome, its role in altering the immune status, and tumor progression is critical.

### 4.1. Microbiome and Neuroblastoma

#### 4.1.1. Gut Microbiome Predicts the Risk for NB

**Figure 3:**
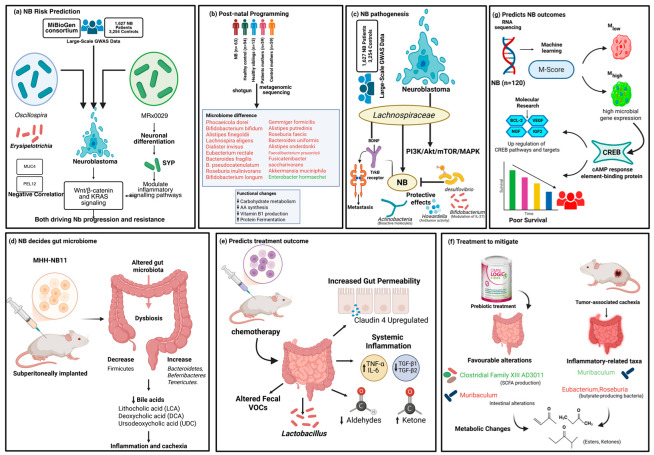
Illustrations depict the following: (**a**) Gut microbiome predicts the risk for NB; (**b**) Postnatal (and not maternal transmission) programming of microbiome in NB patients; (**c**) Function of gut microbe on NB pathogenesis; (**d**) Presence of NB alters gut microbiome composition; (**e**) Microbial composition predicts treatment outcome in NB (murine model); (**f**) Prebiotic treatment mitigate NB-induced microbial mayhem; (**g**) Microbial composition in TME predicts NB outcome.

By applying a Mendelian randomization (MR) approach, J. Chu investigated the casual relationship between gut microbiota and NB [251]. The study was based on large-scale genome-wide association study (GWAS) data from two reputable sources: the MiBioGen consortium for gut microbiota composition and the IEU Open GWAS Project for NB cases, which comprised 1627 children with NB and 3254 controls. Employing the MR method, the author discovered that a higher genetically proxied abundance of the genus *Oscillospira* predicted a higher risk for NB. Notably, the bacterial class *Erysipelotrichia* was associated with a low risk of NB in children, suggesting a possibly protective effect. Erysipelotrichia, recognized for producing SCFAs such as butyrate, has been associated with human health and lipid metabolism. Specifically, MRx0029—a gut bacterium in this category—induces neuronal differentiation in NB cells by fatty acid production, hence elevating MAP2 and SYP levels. These findings support *Erysipelotrichia’s* protective role in NB, possibly due to its influence on neuronal differentiation. Conversely, the genus *Oscillospira* was associated with an increased risk that contributes to NB pathogenesis and according to their composition, gut microbiota may either suppress or promote the proliferation of NB. While this MR-based approach offers a novel, genetically informed perspective, it complements rather than replaces conventional methods such as observational epidemiology, molecular biology, and clinical risk stratification. By leveraging genetic variants as instrumental variables, MR mitigates confounding and reverse causation, thereby enhancing the robustness of etiological insights into NB. To investigate the genetic pathways, Chu identified two significant single nucleotide polymorphisms (SNPs)—MUC4 and PELI2—linked to *Erysipelotrichia*. These two significant genes were specifically associated with NB regulatory genes and were enriched in various upstream transcription factors and multiple tumor progression-related pathways. MUC4 was found to be negatively correlated with FGFR1, an oncogene in NB. PELI2, an E3 ubiquitin ligase, modulates inflammatory signaling pathways including NFκB and MAPK. Both genes were linked to cancer-associated Wnt/β-catenin and KRAS signaling pathways, known to drive NB progression and resistance (Figure 3a).

#### 4.1.2. Postnatal (And Not Maternal Transmission) Programming of Microbiome in NB Patients

To uncover the microbiological alterations in NB, Valles-Colomer and colleagues (2024) conducted a comprehensive metagenomic study of children diagnosed with NB [252]. A study involving 288 individuals utilized shotgun metagenomic sequencing to analyze stool samples from 63 newly diagnosed NB patients, 94 healthy controls, 13 healthy siblings, and 59 mothers from each of the patient and control groups. The findings revealed that children with NB had a significantly lower microbial richness and decreased relative abundance of 18 species including bacteria with reported anti-inflammatory properties such as *P. dorei*, *Bifidobacterium*, and butyrate-producers (*Roseburia*, *Faecalibacterium* spp.) (Figure 3b). In contrast, Enterobacter hormaechei, a potentially pathogenic species, was more prevalent. Functionally, the gut microbiome of these patients showed decreased potential to metabolize carbohydrates (including starch and glycogen), amino acid synthesis (tyrosine and phenylalanine), and vitamin B1 production, along with a shift toward enhanced protein fermentation. These microbiome differences were not observed in healthy siblings or in the mothers of patients, and mother-to-child microbial transmission rates were comparable between all groups. This suggests that alterations in the microbiome of NB patients are likely to occur postnatally and are not attributable to maternal microbial transmission. These findings facilitate further investigation into the potential of microbiome-targeted interventions—such as next-generation probiotics (e.g., *P. dorei*, *Bifidobacterium*) or fecal microbiota transplantation—as adjunctive therapy for NB.

#### 4.1.3. Function of Gut Microbe on NB Pathogenesis

Zhang and colleagues (2024) explored the potential causal link between the gut microbiota and NB through a comprehensive bidirectional Mendelian randomization (MR) analysis and meta-analysis [253]. Using large-scale GWAS data from the IEU Open GWAS Project, they analyzed genetic variants associated with the abundance of 196 gut microbial taxa across several taxonomic levels and evaluated their possible causal impacts on NB risk in a cohort comprising of 4881 people (1627 NB patients and 3254 controls). The MR analysis identified six gut microbiota that exhibited a significant causal relationship with NB. Based on their functional associations, these taxa can be broadly categorized into two functional groups: risk-promoting microbiota, such as *Lachnospiraceae*, and protective microbiota, including *Actinobacteria*, *Bifidobacteriaceae*, *Bifidobacterium*, *Desulfovibrio*, and *Howardella*. Among them, *Lachnospiraceae* is the primary risk factor for NB due to the positive correlation with the expression of BDNF, which is essential for the establishment of peripheral sympathetic and neural crest-derived sensory neurons and promotes metastasis via the TrkB receptor and invades NB via PI3K/Akt/mTOR and MAPK pathways. Conversely, five microbial taxa—Actinobacteria, Bifidobacteriaceae, Bifidobacterium, Desulfovibrio, and Howardella—were found to have protective effects (Figure 3c). *Actinobacteria* and their bioactive molecules’ extracts from 24 of 90 strains isolated from big algae exhibited their ability to decrease NB cell proliferation and reduce cell viability. Bifidobacterium, a well-known probiotic, was shown to inhibit NB-associated pathways potentially through galactose production, modulation of immune cytokine IL-27, and reduction of the PI3K/Akt/mTOR signaling cascade. The genus *Desulfovibrio*, which stimulates the production of hydrogen sulfide (H_2_S), may contribute to the prevention or deceleration of NB progression, as N-acetyl-L-cysteine (NAC), a precursor of hydrogen sulfide (H_2_S), has been shown to inhibit NB cell proliferation. The genus *Howardella*, although less understood, possess anti-tumor activity. These findings not only deepen the knowledge of gut microbial effects on NB pathogenesis but also provide new possibilities for microbiota-targeted preventative and treatment approaches in NB control.

#### 4.1.4. Presence of NB Alters Gut Microbiome Composition

Castellani and colleagues (2017) examined how NB significantly alters the intestinal microbiome and other gut-associated systems, highlighting a direct connection between cancer and gut microbial composition [254]. To investigate this, the human NB cell MHH-NB11 was subperitoneally implanted into immunocompromised mice and compared with the control groups that received only culture medium. Ten weeks after tumor induction, a comprehensive analysis including tumor growth quantification, adipose tissue assessment, measurement of circulating hormones and cytokines, bile acid profiling in serum and feces, and 16S rDNA sequencing of ileal contents revealed significant alterations in the intestinal microbiota of the treatment group compared to the control. Animals with tumors exhibited a decrease in *Firmicutes* and an increase in *Bacteroidetes*, *Beferribacteres*, and *Tenericutes*. Although the differences in microbial populations were not significant, a decrease in *Ruminococcus*, *Dehalobacterium*, and the S24-7 group (*Bacteroidales* family) were observed pointing to gut dysbiosis in tumor-bearing animals. This suggests that NB induces a unique microbial signature, possibly driven by tumor-specific metabolic or immunological changes. The research additionally identified reduced levels of microbiota-derived bile acids in fecal and serum samples from NB mice, including the secondary bile acids lithocholic acid (LCA) and deoxycholic acid (DCA), together with the tertiary bile acid ursodeoxycholic acid (UDC). This reduction indicates dysfunction in gut microbiota which exacerbates inflammation and cachexia in NB (Figure 3d). This demonstrates that NB itself can reciprocally alter the gut microbial environment, likely through tumor-induced metabolic and immunological changes. This perspective is critical for understanding the dynamic interplay between host and microbiota in cancer and supports the rationale for microbiome-targeted interventions aimed at mitigating disease-associated dysbiosis, inflammation, and cachexia.

#### 4.1.5. Microbial Composition Predicts Treatment Outcome in NB (Murine Model)

The relationship between NB, chemotherapy, and gut health has been examined by Castellani and colleagues (2019), who investigated the impact of these factors on metabolism, the fecal microbiome, VOCs, and gut barrier function in a murine model [255]. The human NB cells were implanted into athymic mice, which are immune-deficient, and then these animals were treated with cyclophosphamide (CTX), a common chemotherapy agent used for NB. The presence of a tumor, both independently and in conjunction with chemotherapy, modified the gut microbiota makeup, resulting in increased gut permeability and the induction of systemic inflammation. NB-bearing mice exhibited a decrease in the relative abundance of *Lactobacillus*, which was further diminished by CTX therapy. The decline of Lactobacillus was associated with elevated levels of pro-inflammatory cytokines (TNFα, IL-6) and a concurrent decrease in anti-inflammatory markers (TGF-β1 and TGF-β2), contributing to a catabolic condition and development of cachexia. Additionally, an increased gut permeability, which allowed the harmful substances to leak into the bloodstream, was observed. Both the existence of NB and the chemotherapy treatment in this study seemed to damage this barrier, hence increasing its permeability and elevating the likelihood of systemic inflammation. Furthermore, despite the disruption of gut barrier integrity characterized by increased epithelial apoptosis and FITC-dextran leakage, the tight junction protein claudin 4 was unexpectedly upregulated, suggesting that cell death, rather than tight junction impairment, was likely responsible for the barrier dysfunction. The study also investigated fecal VOCs as indicators of microbial metabolism and identified tumor-associated differences in VOC profiles, characterized by reduced levels of certain aldehydes and elevated ketones, suggesting tumor-induced abnormalities in microbial metabolism (Figure 3e).

#### 4.1.6. Prebiotic Treatment Mitigates NB-Steered Microbial Mayhem

Obermüller and colleagues (2020) demonstrated that prebiotic treatment with OMNi-LOGiC^®^ FIBRE in a mouse model of NB-induced tumor-associated cachexia (TAC) resulted in significant alterations in gut microbiota and metabolic profiles [256]. This prebiotic, composed of dextrin and partially hydrolyzed guar gum, induced a favorable alteration in microbial composition, notably enhancing Clostridial Family XIII AD3011, known for SCFA production, while reducing *Muribaculum*, associated with intestinal inflammation. The microbial alterations were associated with significant changes in fecal VOCs, indicating that the prebiotic influenced microbial metabolism. In TAC mice, inflammatory-related taxa such as *Muribaculum* were elevated, whereas crucial butyrate-producing bacteria like *Eubacterium* and *Roseburia* were diminished.

In parallel, the study also revealed distinct alterations in microbial community. Esters and ketones are the most predominant class affected by NB-associated TAC, with significantly lower quantities of acetone, methylvinylketone, 3-pentanone, and 2-methyl-3-hexanone. These reductions are closely associated with host metabolic stress, particularly in lipid and energy metabolism, as these substances are typically byproducts of microbial fermentation and host metabolic processes involved in fat and amino acid degradation. The results underscore a complex bidirectional connection between NB and the gut microbiome: the malignancy disrupts gut barrier integrity and microbial equilibrium, while the microbiota subsequently alters metabolic and inflammatory responses. Though the prebiotic by itself did not correct the cachexia condition, its capacity to alter the microbiome positively suggests potential for future therapeutic strategies targeting the gut microbiota to support NB patients (Figure 3f).

#### 4.1.7. Microbial Composition in TME Predicts NB Outcome

Li. X and colleagues (2022) explored the intriguing relationship between the tumor-associated microbiome and patient outcomes in NB [257]. This research analyzed RNA sequencing data from 120 tumor samples to determine if microbial gene expression in the tumor microenvironment could serve as a novel prognostic marker. Utilizing machine learning methodologies, they developed a Microbial Gene Abundance Score (M-score), which reflects the relative activity of microbial genes within the tumor microenvironment. This stratification revealed two distinct subgroups among high-risk patients: M_high (high microbial gene expression) and M_low (low microbial gene expression). Patients in the M_high group exhibited significantly poor overall and event-free survival compared to those in the M_low group, suggesting that microbial gene expression offers prognostic insights beyond the traditional Children’s Oncology Group (COG) risk classification. Molecular research indicated that tumors in the M_high group exhibited an upregulated activity in specific pathways in NB progression. Particularly, the upregulation of the CREB (cAMP response element-binding protein) pathway and its oncogenic targets, including BCL-2, VEGF, NGF, and IGF2, all of which are known to promote tumor cell survival, angiogenesis, and metastasis, was observed (Figure 3g). These data indicate that microbiomes may actively influence tumor biology and disease progression rather than just coexisting inside the tumor.

## 5. Clinical Research Landscape: Challenges and Ongoing Trials in Neuroblastoma Therapy

Despite the evidence implicating the microbiome in the pathogenesis and therapeutic responsiveness of NB, several critical challenges impede its clinical translation. A foremost concern is the heterogeneity in microbiome composition, influenced by variables such as age, dietary habits, antibiotic exposure, and geographic factors. This variability complicates the identification of consistent microbial biomarkers that could reliably predict NB risk or therapeutic outcomes. Furthermore, the predominance of cross-sectional studies limits the capacity to elucidate temporal dynamics and causal relationships between microbiome alterations and disease progression. The paucity of pediatric-specific microbiome research further constrains translational applicability, as developmental differences in immune system maturation and microbial colonization render extrapolation from adult oncology data problematic. Additionally, the intricate bidirectional interactions among the host, microbiome, and tumor microenvironment encompassing immune modulation, metabolic reprogramming, and epigenetic regulation pose significant analytical challenges in delineating mechanistic pathways. From a clinical implementation standpoint, the absence of standardized methodologies for microbiome sampling, sequencing, and bioinformatic analysis, coupled with ethical and regulatory concerns surrounding interventions such as FMT in pediatric populations, further complicates integration into routine clinical practice (Table 8)**.**

## 6. Conclusions

The human microbiome plays a pivotal role in cancer biology, influencing tumor initiation, progression, immune response, and treatment outcomes. Across multiple malignancies, microbial communities have been shown to promote or inhibit carcinogenesis by altering inflammation, metabolic pathways, and immune surveillance. Despite considerable advancements in understanding the role of microbial imbalances in inflammation, metabolic dysregulation, and immune evasion across several malignancies, NBs’ diversity, function, and mechanism are relatively less explored. Emerging evidence continues to reveal the diverse mechanisms through which microbiota affect host physiology, including epigenetic regulation, metabolite production, and interactions with signaling pathways involved in NB oncogenesis.

Considering NB’s origin from NCCs and its dynamic interplay with the immune system, the potential for microbial manipulation of its tumor microenvironment is significant. The available data suggests a complex bidirectional relationship—wherein NB influences microbial composition, and in turn, the altered microbiome may modulate immune responses, inflammatory signaling, and systemic metabolism in ways that could affect tumor growth and therapy outcomes. Further investigations on analyzing the temporal and molecular relationships between microbial alterations in NBs’ genesis, specifically how early-life microbiota disruptions may predispose certain children to NB or affect their immune cell type composition, are needed. Innovative methodologies, like longitudinal microbiome monitoring, gnotobiotic animal models, and high-resolution single-cell sequencing of tumor-associated microbial genes, can elucidate these mechanisms. Moreover, the realization of the therapeutic potential of microbiota alteration for NB cure is still in its early stages. Likewise, understanding the mode of action of the gut and/or tumor microbiome during disease progression and NB evolution is critical. Strategies such as targeted probiotic supplementation, dietary modifications, and microbial metabolite modulation offer promising adjuncts to existing treatments. These approaches may help recalibrate the immune milieu, enhance treatment efficacy, and reduce adverse effects in vulnerable pediatric populations. These critical gaps in knowledge not only underscore the unrealized realm of NB biology but also highlight the immense potential for breakthroughs for the cure of this deadly disease. Exploring how microbial communities influence NB initiation, progression, and therapeutic response may uncover transformative opportunities for personalized medicine, enabling more precise prognostic tools and innovative microbiome-based therapies.

## Figures and Tables

**Table 1:** Evolution of different microbial communities across the human lifespan, from birth to adulthood.

Stage	Microbiome Development	Influencing Factors
Birth	Colonization commences with germs originating from the maternal body (vagina, feces, skin).	Method of delivery (vaginal versus caesarean)
Newborn infant (1–4 weeks)	Initially dominated by Staphylococcus and Enterobacteriaceae, followed by subsequent succession by Bifidobacterium.	Feeding technique (breast milk versus formula), gestational age, antibiotic administration
Infancy (2 years)	Enrichment of Bifidobacterium and incorporation of lactic acid bacteria	Duration of breastfeeding, adoption of solid meals
Childhood (2–4 years)	Change to mature microbes, rise of Bacteroides	Environmental exposures
Adulthood (above 18 years)	Firmicutes and Bacteroidetes	Lifestyle, nutrition, environmental conditions

**Table 5:** International Neuroblastoma Risk Group Staging System (INRGSS).

INRGSS	Features	Risk Groups	Event-Free Survival
L1	Locoregional tumor without any identified risk factors based on imaging	Very low—low risk	5-year—>75–85%
L2	Tumor cells have metastasized to adjacent tissues	Low risk	5-year—75–85%
M	NB cells spread to distant organs	Low risk—high risk	5-year—50–75%
MS	Metastatic disease localized to the skin, liver, or bone marrow.	High risk	5-year—<50%

**Table 6:** International Neuroblastoma Staging System (INSS).

INSS Stage	Description	Risk Group	5-Year Survival Rate (%)
Stage 1	Localized tumor, completely resected by surgery	Low Risk	90–95%
Stage 2A	Tumor localized but cannot be completely removed by surgery	Low/Intermediate Risk	80–90%
Stage 2B	Tumors on one side may not always be fully resectable	Intermediate Risk	75–85%
Stage 3	Unresectable tumor that may involve lymph nodes but has not spread distantly	High Risk	50–70%
Stage 4	Cancer has spread to distant sites (e.g., bone, liver, bone marrow)	High Risk	20–40%
Stage 4S	In children <1 year, cancer has spread to liver, skin, and/or bone marrow (≤10% involvement)	Low/Intermediate Risk	80–95%

**Table 7:** Categorizes tumor microenvironment (TME) subtypes in high-risk NB based on immune activity and gene expression profiles [250]. Each subtype is associated with distinct biological features, such as neoantigen load and oncogenic signaling, which influence patient survival outcomes.

TME Subtype	Immune Characteristics	Genomic Features	Pathway Enrichment	Clinical Implication
T cell-inflamed	High CD8+ T cell infiltration, IFN- γ signature, immune checkpoint molecules	High neoantigen load, diverse TCR repertoire	Immune-related pathways (e.g., IFN signaling)	Best overall and event-free survival
Intermediate	Moderate immune cell markers	Variable neoantigen burden	Moderate immune and oncogenic signaling	Intermediate prognosis
Non-T cell-inflamed	Low immune gene expression, T cell exclusion signatures	Activation of MYCN, ASCL1, SOX11, KMT2A, even without MYCN amplification	Neurodevelopmental and cell cycle pathways	Poor survival; resistant to immunotherapy

**Table 8:** Comprehensive summary of ongoing clinical trials in high-risk NB.

Trial Title	Description	Eligibility Criteria	Objective	Lead Organization	Phase
Dinutuximab with Chemo-therapy, Surgery and Stem Cell Transplantation for the Treatment of Children with Newly Diagnosed High Risk NB	Tests the addition of dinutuximab to induction chemotherapy and standard care in high-risk NB	≤30 years, newly diagnosed high-risk NB, specific renal/liver/cardiac function criteria	To determine if early chemoimmunotherapy improves event-free survival	Children’s Oncology Group	Phase III
Eflornithine (DFMO) and Etoposide for Relapsed/Refractory NB	DFMO + etoposide in relapsed/refractory NB	≤30.99 years, relapsed/refractory NB, prior multi-drug chemotherapy	Evaluate safety and efficacy of DFMO + etoposide	Giselle Sholler	Phase I/II
A Study of Therapeutic Iobenguane (131-I) and Vorinostat for Recurrent or Progressive High-Risk NB Subjects	131I-MIBG + Vorinostat for recurrent/progressive NB	Iobenguane-avid high-risk NB, prior induction therapy, stem cell availability	Evaluate efficacy and safety of combination therapy	DRAXIMAGE	Phase II
A Study of a Vaccine in Combination with Beta-glucan in People with NB	OPT-821 vaccine + beta-glucan for high-risk NB	HR-NB in CR, ≥21 and ≤180 days post systemic therapy, adequate organ function	Assess anti-GD2 antibody titers	Memorial Sloan Kettering Cancer Center	Phase II
Naxitamab Added to Induction for Newly Diagnosed High-Risk NB	Naxitamab added to 5 cycles of induction chemotherapy	≤21 years, newly diagnosed high-risk NB, specific INSS stages	Evaluate efficacy and safety of naxitamab in induction	Giselle Sholler	Phase II
Autologous hALK. Chimeric Antigen Receptor T Cells (hALK.CAR T) for the Treatment of Relapsed or Refractory High-Risk NB	hALK.CAR T cell therapy for relapsed/refractory NB	≥12 months and <30 years, relapsed/refractory high-risk NB	Identify MTD and assess safety and efficacy	Dana-Farber Harvard Cancer Center	Phase I/II
67Cu-SARTATE™ Peptide Receptor Radionuclide Therapy Administered to Pediatric Patients With High-Risk, Relapsed, Refractory NB	Adaptive trial of 67Cu-SARTATE in pediatric high-risk NB	High-risk NB, adequate organ function, stem cell product available	Evaluate safety and efficacy of 67Cu-SARTATE	Clarity Pharmaceuticals	Phase I/II
Donor Immune Cells (Allogenic Ex Vivo Expanded Gamma Delta T Cells), Dinutuximab, Temozolomide, Irinotecan and Zoledronate for the Treatment of Refractory, Relapsed, or Progressive NB or Osteosarcoma in Children	Gamma delta T cells + dinutuximab + chemo for refractory/relapsed NB	≥12 months, high-risk NB or osteosarcoma, measurable disease	Determine MTD and define toxicities	Emory University Hospital	Phase I
Reduced Chemotherapy (N10) for the Treatment of High-Risk NB in Children	N10 chemo regimen for high-risk NB	<19 years, HR-NB, ≤1 prior HR-NB chemo cycle	Assess early CR rate and survival outcomes	Memorial Sloan Kettering Cancer Cente	Phase II
High Risk NB, a Study 1.8 of SIOP-Europe (SIOPEN)	Multimodal treatment protocol with randomized immunotherapy arms	High-risk NB (stages 2–4s, MYCN+ or >12 months)	Improve EFS with BuMel MAT and immunotherapy, including immunotherapy (e.g., IL-2) which may interact with gut microbiome.	St. Anna Kinderkrebsforschung	Phase I/II

## Data Availability

All data and materials are available in the main text of the manuscript.

## List of Abbreviations

SCFAs	Short-chain fatty acids
HMOs	Human milk oligosaccharides
VOCs	Volatile organic compounds
IBD	Inflammatory bowel disease
TME	Tumor microenvironment
EBV	Epstein–Barr virus
HTLV	Human T cell lymphotropic virus
CRC	Colorectal cancer
TLRs	Toll-like receptors
BFT	Bacteroides fragilis toxin
NFκB	Nuclear factor kappa light chain enhancer of activated B cell
HDAC	Histone deacetylases
GC	Gastric cancer
CagAA	Cytotoxin-associated gene
VacA	Vacuolating cytotoxin A
CNS	Central nervous system
ICB	Immunological checkpoint blockade
NOCs	N-nitroso Compounds
DCA	Deoxycholic acid
HCC	Hepatocellular carcinoma
ROS	Reactive oxygen species
RNS	Reactive nitrogen species
TNFα	Necrosis Factor-alpha
IL-6	Interleukin-6
IL-1β	Interleukin-1 beta
STAT3	Signal transducer and activator of transcription 3
Tregs	Regulatory T cells
PD-1	Programmed Cell Death Protein 1
CTLA-4	Cytotoxic T-Lymphocyte Antigen 4
ALL	Acute lymphoblastic leukemia
GALT	Gut-associated lymphoid tissue
BDNF	Brain-derived neurotrophic factor
LPS	Lipopolysaccharides
BBB	Blood-brain barrier
IL-10	Interleukin-10
HL	Hodgkin’s lymphoma
NHL	Non-Hodgkin’s lymphoma
NCC	Neural crest cells
NB	Neuroblastoma
IgA	immunoglobulin A
HAIs	Hospital-associated infections
GOS	Galactooligosaccharides
FOS	Fructooligosaccharides
IMCT	Intensive multi-modal clinical therapy
INRGSS	International Neuroblastoma Risk Group Staging System
INSS	International Neuroblastoma Staging System
DC	Dendritic cell
MHC-I	Major histocompatibility complex class I
TGF-β	Transforming growth factor-beta
PGE2	Prostaglandin E2
IFN-γ	Interferon-gamma
NKG2D	Natural killer group 2 member D
GWAS	Genome-wide association study
SNPs	Single nucleotide polymorphisms
MR	Mendelian randomization
LCA	Lithocholic acid
DCA	Deoxycholic acid
UDC	Ursodeoxycholic acid
CTX	Cyclophosphamide
TAC	Tumor-associated cachexia
COG	Children’s Oncology Group
CREB	cAMP response element-binding protein
